# IMPACT: A web server for exploring immunotherapeutic predictive and cancer prognostic biomarkers

**DOI:** 10.1002/ctm2.1354

**Published:** 2023-08-30

**Authors:** Yutao Liu, Yundi Zhang, Wenchuan Xie, Jing Zhao, Yiting Dong, Chunwei Xu, Yanan Wang, Man Li, Guoqiang Wang, Xin Zhu, Wenxian Wang, Kequan Lin, Huafei Lu, Yusheng Han, Leo Li, Jianchun Duan, Shangli Cai, Jie Wang, Zhijie Wang

**Affiliations:** ^1^ State Key Laboratory of Molecular Oncology Department of Medical Oncology National Cancer Center/National Clinical Research Center for Cancer/Cancer Hospital Chinese Academy of Medical Sciences and Peking Union Medical College Beijing China; ^2^ CAMS Key Laboratory of Translational Research on Lung Cancer, State Key Laboratory of Molecular Oncology Department of Medical Oncology National Cancer Center/National Clinical Research Center for Cancer/Cancer Hospital, Chinese Academy of Medical Sciences & Peking Union Medical College Beijing China; ^3^ Burning Rock Biotech Guangdong China; ^4^ Institute of Basic Medicine and Cancer (IBMC) Chinese Academy of Sciences Zhejiang China; ^5^ Department of Clinical Trial The Cancer Hospital of the University of Chinese Academy of Sciences (Zhejiang Cancer Hospital) Zhejiang China

Dear Editor,

Identifying reliable biomarkers for immune checkpoint inhibitors (ICIs) can efficiently screen beneficiaries to improve their clinical use. We developed a public web server, IMPACT, to facilitate the comprehensive investigation of predictive or prognostic biomarkers, interaction effects and biological mechanisms with both public and in‐house datasets (http://www.brimpact.cn/ or http://impact.brbiotech.com/) (Figure 1A).

**FIGURE 1:**
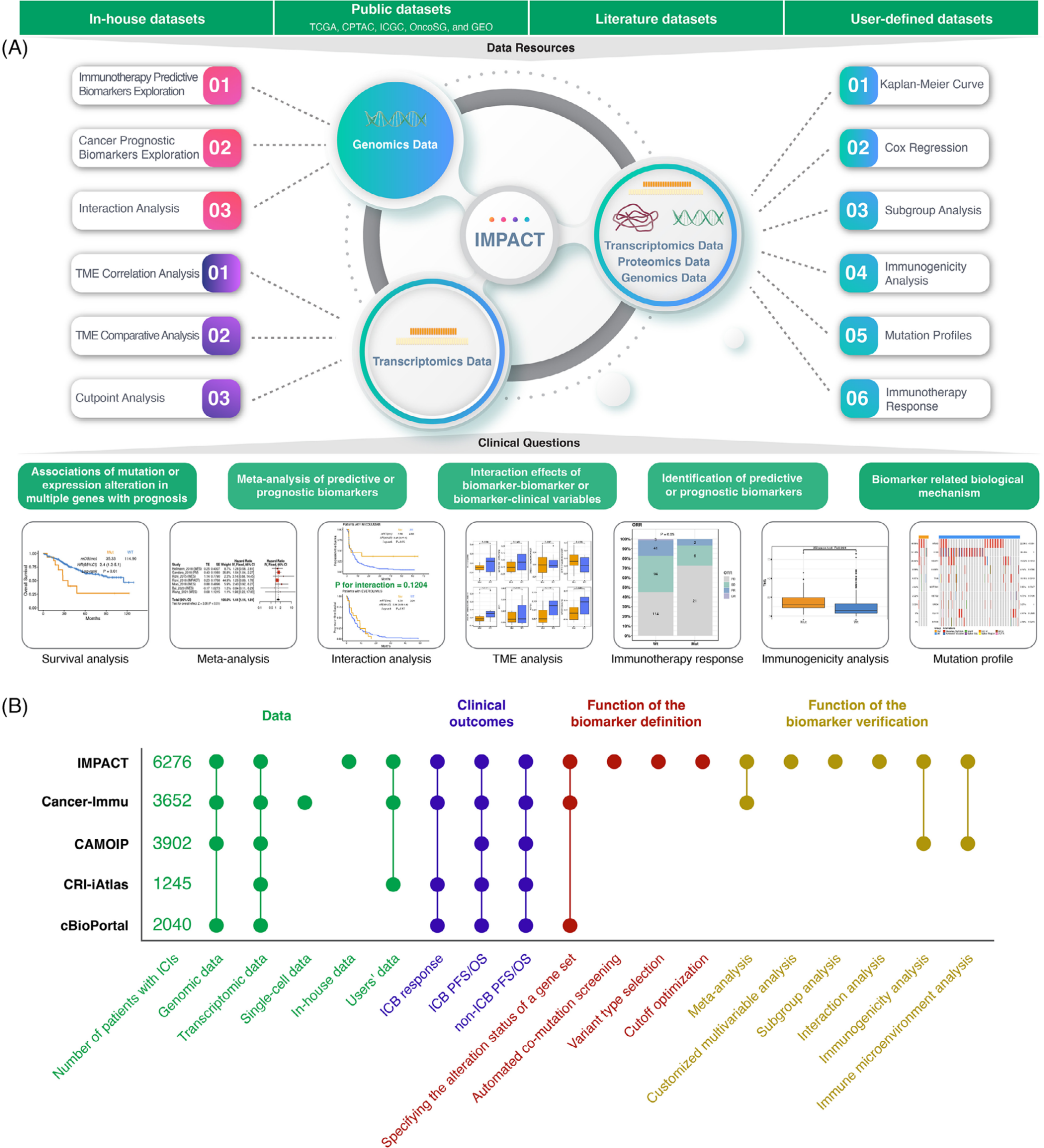
The overview of IMPACT. (A) The diagram of IMPACT. (B) Comparison among IMPACT and existing biomarker exploration tools.

ICIs have heralded a new era in cancer treatment, while most ICI biomarkers have low generalisation performance due to limited data and statistical methods. Currently, several public database‐based tools are available to explore potential biomarkers, such as CAMOIP,^1^ Cancer‐Immu,^2^ CRI‐iAtlas^3^ and cBioPortal.^4^ Compared with these tools (Figure 1B and Table S1), IMPACT uniquely allows users to define customised biomarkers by selecting mutation variant types and automatically screening cut‐points for continuous biomarkers and any mutation or co‐mutation in a gene set and to conduct more rigorous key analyses including customised multivariable, subgroup and interaction analyses in more datasets (Table S2). Here, we illustrate how to use IMPACT to comprehensively explore biomarkers.

Users can start by understanding the general performance of a biomarker using PredExplore and ProgExplore modules to analyse the association between gene alteration and survival across all the ICI and non‐ICI datasets. Detailed results in each dataset and forest plot of meta‐analysis can be automatically generated. Especially, these modules allow users to automatically explore a predictive or prognostic value of any mutation or co‐mutation in a gene set. As a case of DNA damage response (DDR) pathway alteration (Figures 2A,B and S1A,B), the results suggest that pathway mutation could accumulate the minimal effect of single DDR gene mutation to observe a significant association with prolonged survival to ICIs. Co‐mutation is usually used to explore synergistic or mutually exclusive effects between genes. The co‐mutation analysis of *KRAS/TP53/STK11* with IMPACT showed that predictive effects of *KRAS* depended on the presence of other co‐mutation genes, which was consistent with previous results (Figures 2C–F and S1C,D and Table S3).^5^

**FIGURE 2:**
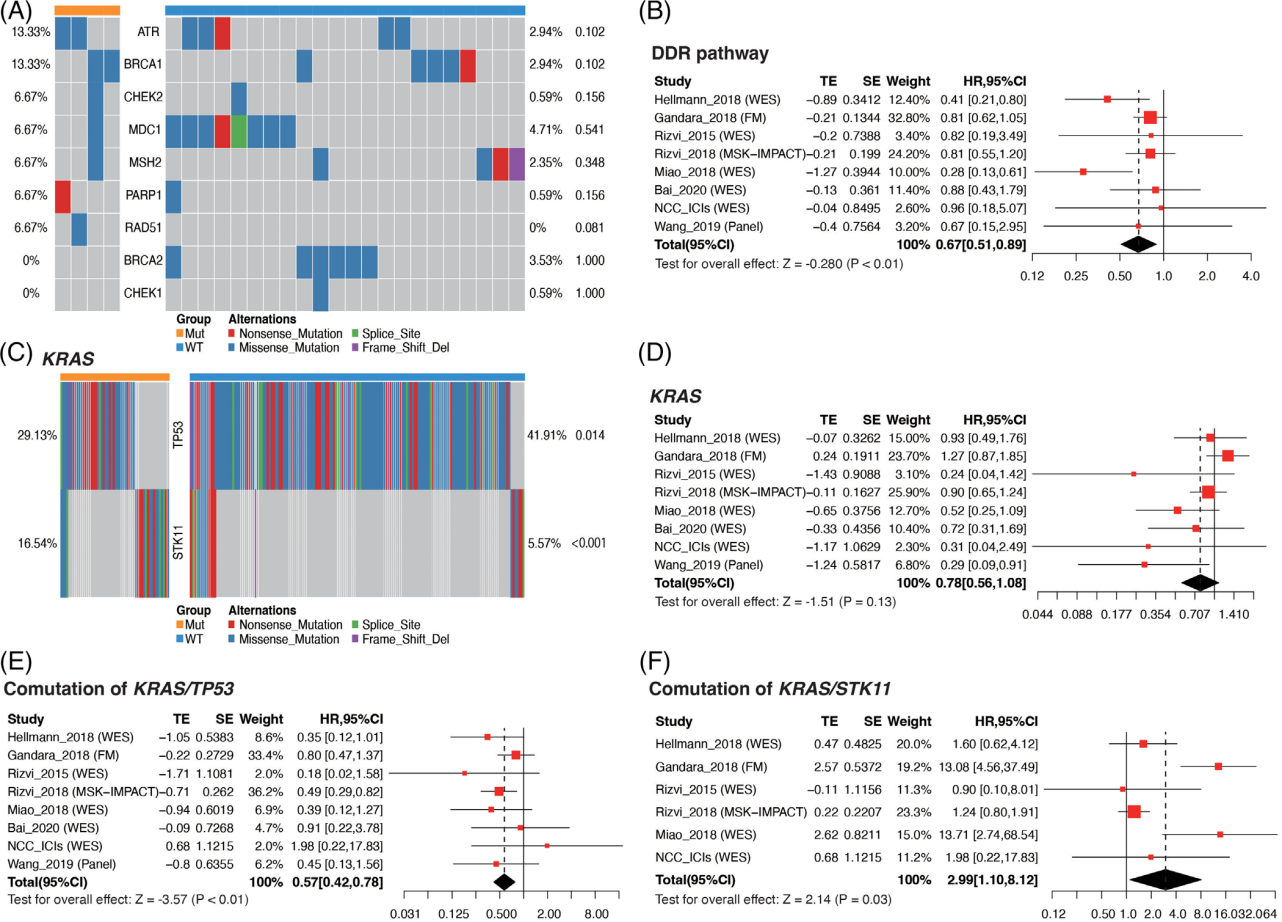
Examples of pathway alteration and co‐mutation analyses. (A) Waterfall plot of DNA damage response (DDR) pathway genes according to *ATM* mutation. (B) Meta‐analysis of associations between DDR pathway alterations and progression‐free survival. (C) Waterfall plot of *STK11* and *TP53* gene mutations according to *KRAS* mutation status. (D) Meta‐analysis of associations between *KRAS* gene mutation and progression‐free survival. (E) Meta‐analysis of associations between *KRAS*/*TP53* co‐mutation and progression‐free survival. (F) Meta‐analysis of associations between *KRAS*/*STK11* co‐mutation and progression‐free survival.

To further explore the biomarker of interest, users can verify the finding with a detailed analysis in each dataset. Compared with similar functions of other tools, the survival analysis module allows users to freely select variant types to define meaningful mutation status, screen cut‐points for continuous variables, and perform customised multivariable and subgroup analyses to verify independence of a biomarker. Here, *PBRM1*, bTMB and *ATM* are used as examples to illustrate these functions: (1) mutation types. Since different types of mutations usually influence gene functions (e.g., gain or loss of function), it is necessary to consider mutation types when defining gene alterations. As reported in kidney cancer, patients with *PBRM1*‐truncating mutations had significantly longer overall survival (OS) than those with *PBRM1* non‐truncating mutations but not non‐synonymous mutations.^6^ The same result was obtained using IMPACT (Figure 3A,B). (2) Continuous biomarkers. For continuous biomarkers, such as gene expression and tumor mutational burden (TMB), it is critical to explore the effects of selected cut‐off values on the associations between biomarkers and survival. Previously, Gandara et al. failed to demonstrate the association of blood‐based TMB and OS with a pre‐defined cut‐off.^7^ Using the cutpoint analysis sub‐module on IMPACT, the forest plot of hazard ratio based on various bTMB cut‐offs showed that bTMB tended to be negatively associated with OS (Figure 3C,D), which inspired a valuable work of using maximum somatic allele frequency modified bTMB to predict ICI outcome.^7^ (3) Verify the independence of biomarkers. To determine whether a biomarker is independent of other factors, multivariable and subgroup analyses can be done in the Cox regression and subgroup analysis sub‐modules. Conveniently, users can freely select confounders and stratified factors according to their prior knowledge rather than a fixed factor (Figures 3E and S2A).

**FIGURE 3:**
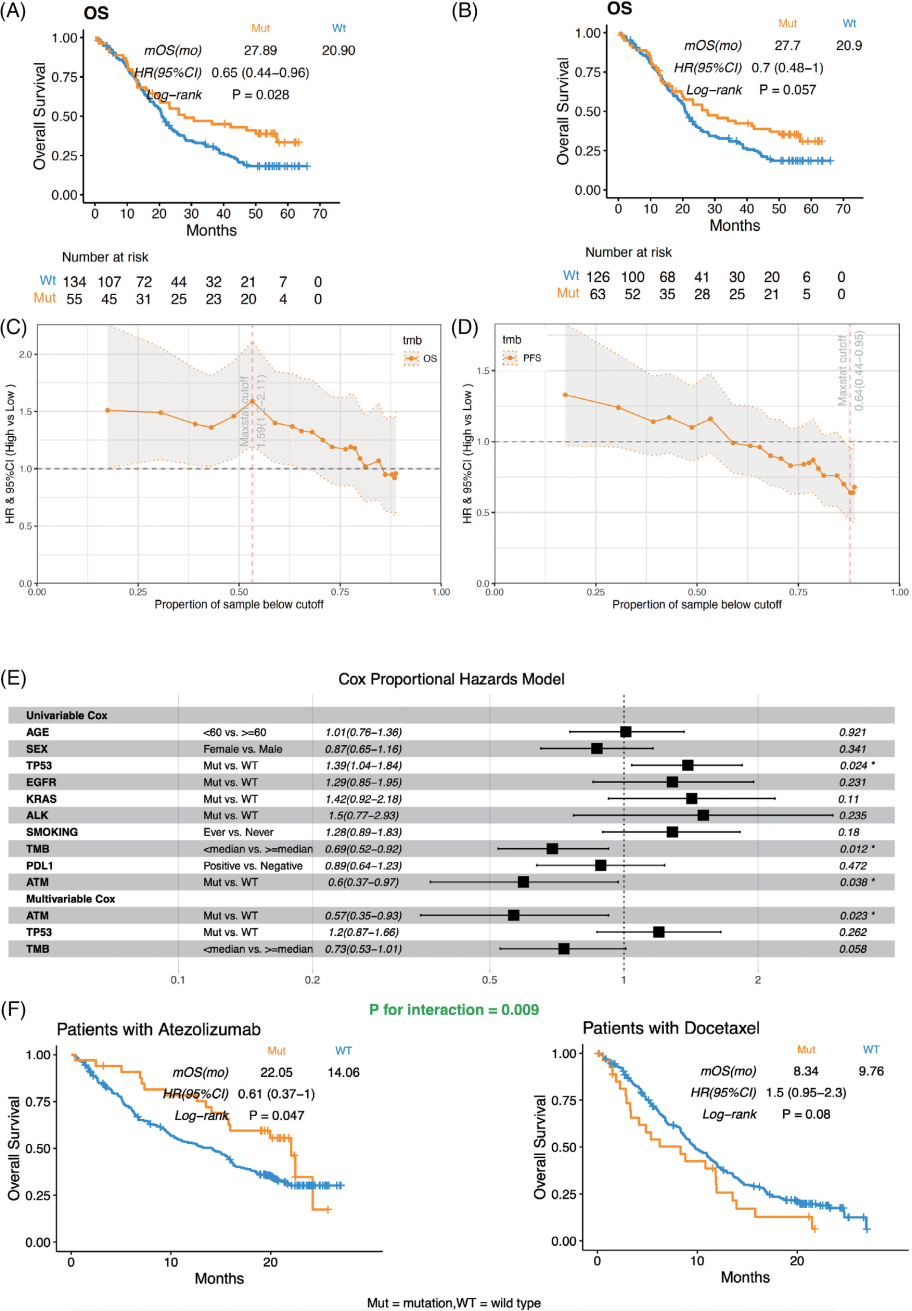
Examples of survival analysis and interaction analysis modules. (A) The association of *PBRM1* truncating mutation with overall survival (OS) in the kidney cancer dataset of Checkmate_025. (B) The association of *PBRM1* nonsynonymous mutation with OS in the kidney cancer dataset of Checkmate_025. (C) The hazard ratios of bTMB and OS across various cutoffs in the Gandara_2018 dataset. (D) The hazard ratios of bTMB and progression‐free survival (PFS) across various cutoffs in the Gandara_2018 dataset. (E) Univariable and multivariable Cox regression of *ATM* mutation with PFS in the Gandara_2018 dataset. (F) Kaplan–Meier curve of *ATM* mutation for overall survival in patients treated with atezolizumab and docetaxel.

Moreover, users can implement interaction analysis module, which is uniquely available on IMPACT, to determine whether a potential biomarker is predictive of immunotherapeutic efficacy by analysing the interaction effects between biomarkers and treatment groups. Here, we use *ATM* and *STK11* to illustrate this function. *ATM* mutation was reported as a predictive biomarker by analysing the ICI databases of lung adenocarcinoma.^8^ However, it was undefined whether it is an ICI‐specific predictive biomarker. The result of interaction analysis module shows that *ATM* mutation was significantly associated with prolonged survival only in patients treated with ICIs, instead of patients with other treatments and the *p*‐value for interaction was significant (*p* for interaction = .009, Figure 3F), indicating that the *ATM* mutation is a predictive biomarker specifically for ICI treatment. In contrast, *STK11* mutation was associated with worse survival in both treatment and control arms (*p* for interaction = .96, Figure S2B), which suggests that the *STK11* mutation is a prognostic biomarker independent of treatment. This result was absent in the previous study.^9^

After a potential biomarker has been identified by the above analysis, immunogenicity and tumor microenvironment (TME) modules provide users with a simple way to analyse immunogenicity or immune signatures, aiding to understand the biological mechanisms. In the immunogenicity module, users can analyse the correlation between gene expression/mutation and TMB, neoantigen and genome instability scores (Figure 4A–C). Moreover, TME analysis module allows users to analyse the correlation between gene expression and oncogenic/immune signatures (Figure 4D–F).

**FIGURE 4:**
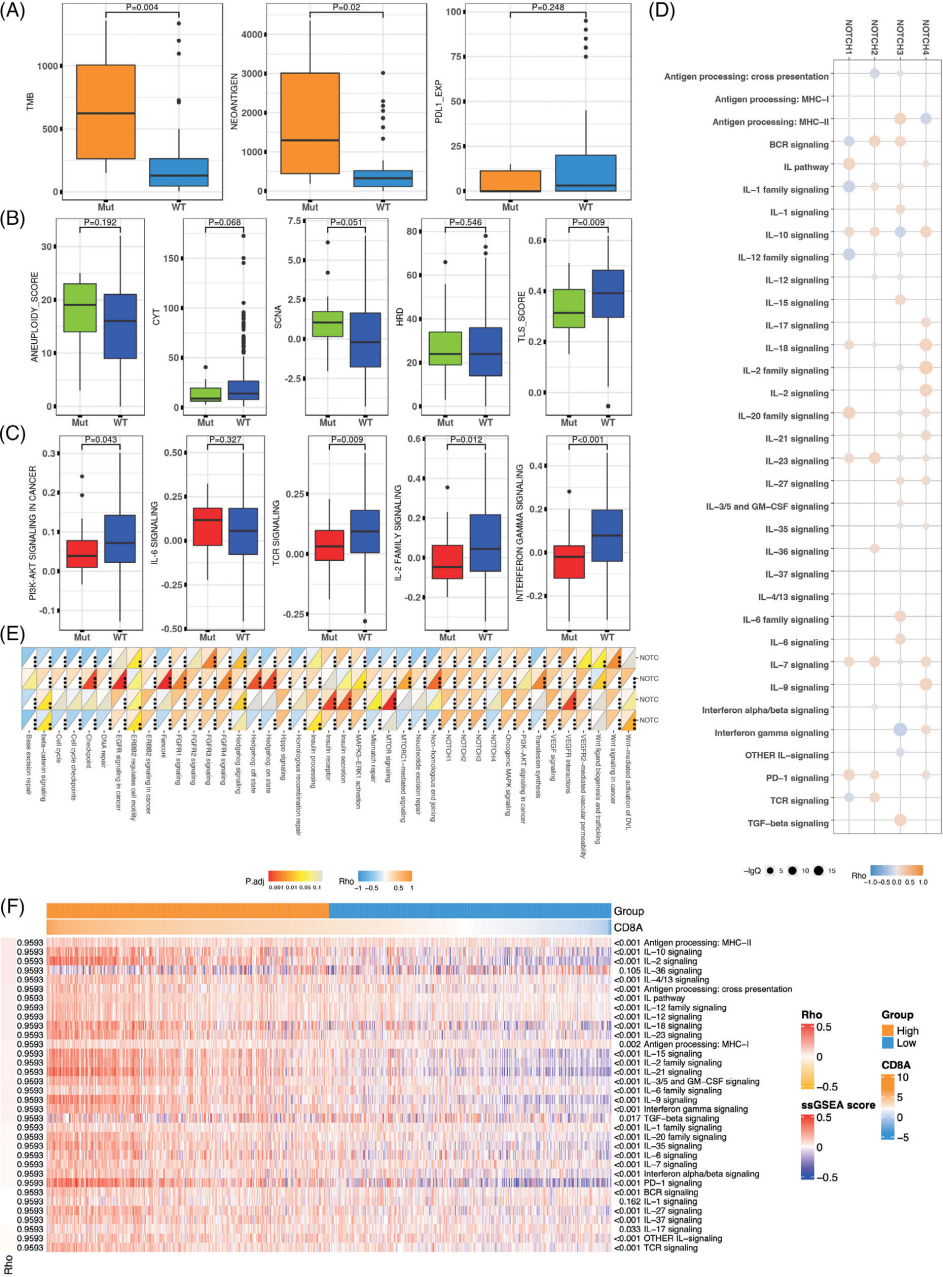
Examples of immunogenicity and tumor microenvironment (TME) analysis modules. (A) The association of ATM nonsynonymous mutation with tumor mutational burden (TMB), neoantigen and programmed cell death protein ligand 1 (PD‐L1) expression in the lung cancer dataset of Hellmann_2018. (B) The association of *ATM* nonsynonymous mutation with genome instability indicators and immune infiltration signatures in the TCGA lung adenocarcinoma (LUAD) dataset. (C) The association of *ATM* nonsynonymous mutations with oncogenic and immune signatures in the TCGA LUAD dataset. (D) The correlation between *NOTCH* pathway expression and immune signatures in the TCGA LUAD dataset. (E) The correlation between *NOTCH* pathway expression and oncogenic signatures in the TCGA LUAD dataset. (F) The heatmap of immune pathway expression between high or low CD8A expression groups in the TCGA LUAD dataset.

Although IMPACT provides comprehensive functions for biomarker exploration (Table S4), further biological or clinical validation is needed to validate the identified biomarkers. Previously, using IMPACT, we noticed a negative association between *TGFBR2* mutation and survival after immunotherapy (Figure S2C). Subsequently, we also reported a case in which a lung cancer patient with a *TGFBR2* mutation experienced hyper‐progression after receiving ICI monotherapy.^10^ These findings suggest that the biomarkers discovered by IMPACT may be validated in the clinic, which shows the potential value of IMPACT in biomarker exploration.

In summary, IMPACT is a user‐friendly platform, conveying a comprehensive resource with more datasets and functions for sophisticated exploration of predictive and/or prognostic biomarkers, interaction effects and potential biological mechanisms, which eases bioinformatic analyses for researchers. With long‐term support, continuous upgrading and optimisation, we believe IMPACT will be a popular tool to facilitate immunotherapy research.

## CONFLICT OF INTEREST STATEMENT

The authors declare they have no conflicts of interest.

## Supporting information

Supporting information

Supporting information

Supporting information

## Data Availability

All the data used in IMPACT can be downloaded from our server at https://impact.brbiotech.com (or http://www.brimpact.cn/). All the code can be accessed at https://github.com/wenchuanxie/IMPACT. All the descriptions of methods can be found in the Supporting Information.
