# Supplementary material for: IMPACT: A web server for exploring immunotherapeutic predictive and cancer prognostic biomarkers
Source: Clin Transl Med. 2023 Aug 30;13(9):e1354. doi: 10.1002/ctm2.1354 (PMC10468578; doi:10.1002/ctm2.1354)
Supplement: Supplementary file 2 — Supporting information [file CTM2-13-e1354-s002.pptx]

## Slide 1
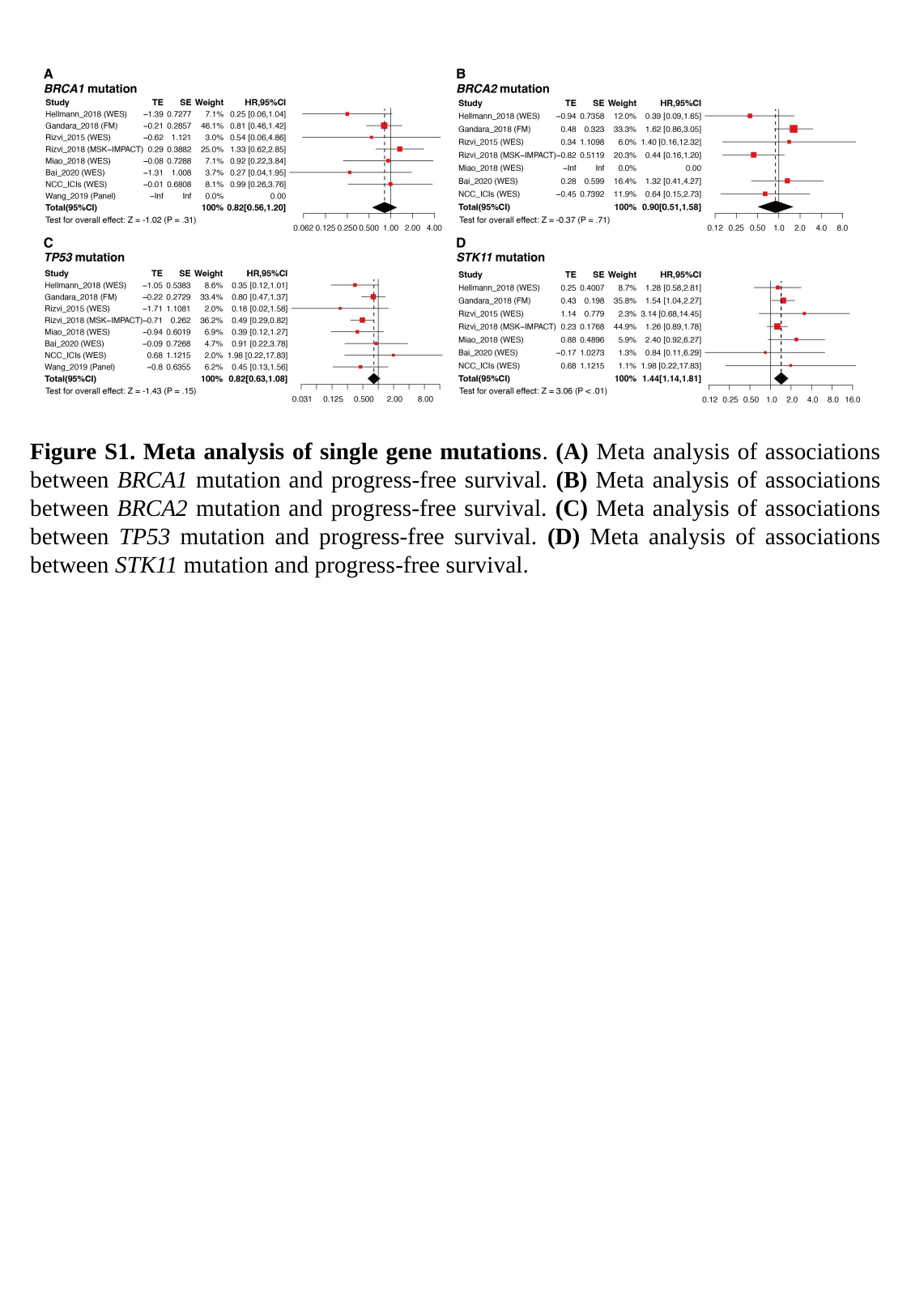

Figure S1. Meta analysis of single gene mutations. (A) Meta analysis of associations between BRCA1 mutation and progress-free survival. (B) Meta analysis of associations between BRCA2 mutation and progress-free survival. (C) Meta analysis of associations between TP53 mutation and progress-free survival. (D) Meta analysis of associations between STK11 mutation and progress-free survival.

## Slide 2
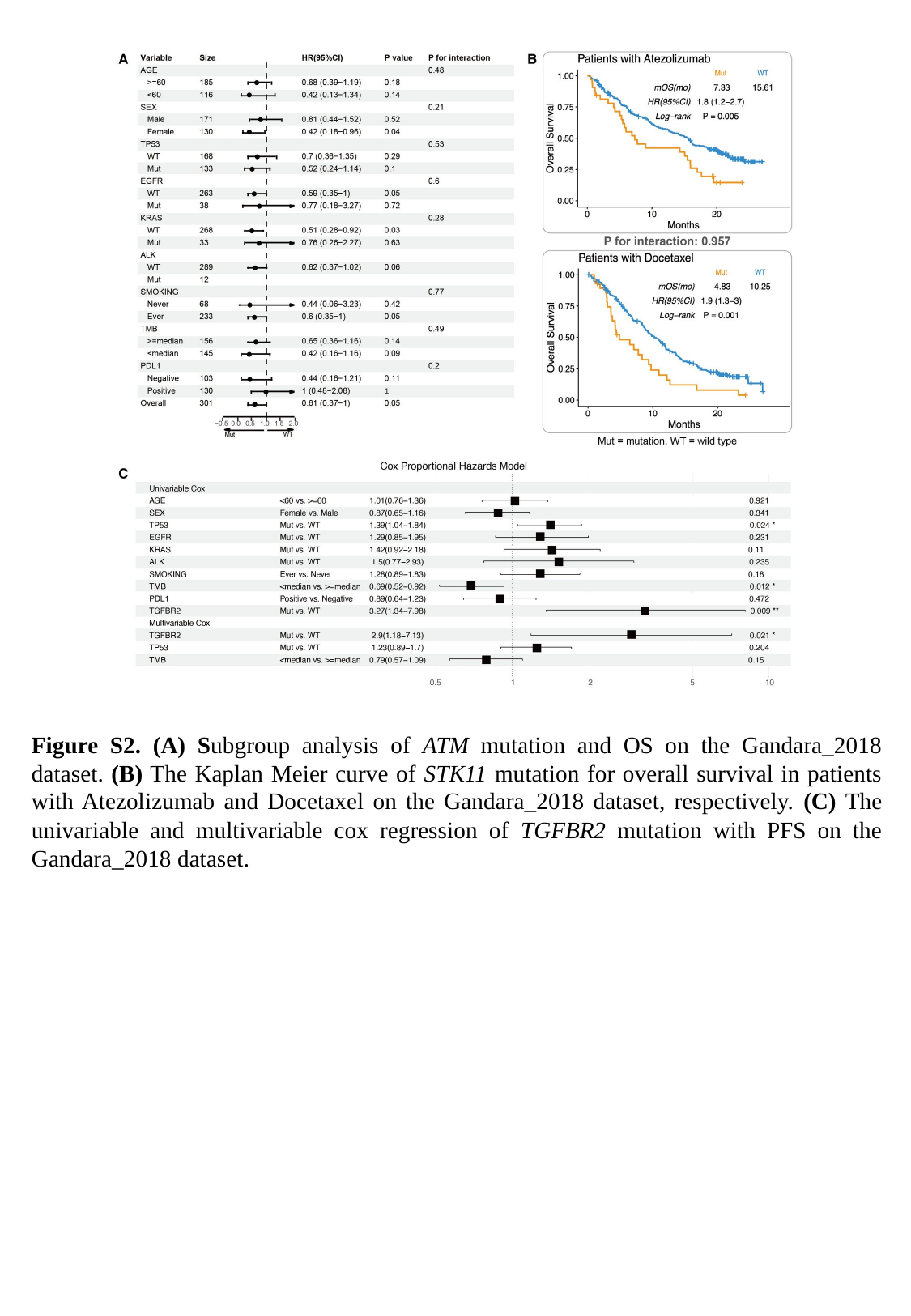

Figure S2. (A) Subgroup analysis of ATM mutation and OS on the Gandara_2018 dataset. (B) The Kaplan Meier curve of STK11 mutation for overall survival in patients with Atezolizumab and Docetaxel on the Gandara_2018 dataset, respectively. (C) The univariable and multivariable cox regression of TGFBR2 mutation with PFS on the Gandara_2018 dataset.
