# Supplementary material for: IMPACT: A web server for exploring immunotherapeutic predictive and cancer prognostic biomarkers
Source: Clin Transl Med. 2023 Aug 30;13(9):e1354. doi: 10.1002/ctm2.1354 (PMC10468578; doi:10.1002/ctm2.1354)
Supplement: Supplementary file 3 — Supporting information [file CTM2-13-e1354-s003.docx]

**Supplemental Methods**

**Overview of IMPACT**

IMPACT was developed to systematically investigate predictive or prognostic biomarkers, relevant interaction effects, and biological mechanisms for immunotherapy (**Figure 1**). Seven function modules are implemented in IMPACT, including PredExplore (Immunotherapy Predictive Biomarker Exploration), ProgExplore (Cancer Prognostic Biomarker Exploration), Survival Analysis (composed of Kaplan-Meier Curve, Cox Regression, Subgroup analysis, Cutpiont Analysis, Immunotherapy Response), Interaction Analysis, Immunogenicity Analysis, Microenvironment Analysis, and Mutation Profiles.

IMPACT is implemented using Shiny (https://shiny.rstudio.com/). MySQL was used as a backend database to store clinical information, omics datasets and external metadata. To improve the performance of IMPACT, the processed input data was stored in cache as R object files.

**Data collection**

The genomic or transcriptomic data of patients treated with ICIs were collected from 24 public datasets and 3 in-house datasets, corresponding to 6,276 samples across 10 cancer types (**Supplementary Table S1**). In addition, 1 in-house non-ICI dataset and 48 public non-ICI datasets were curated from databases of TCGA, GEO, CPTAC, ICGC, and OncoSG. In detail, the genomic data were generated based on the whole exome sequencing (WES) or the next-generation sequencing (NGS) panel, while the transcriptomic data were derived from the whole transcriptome sequencing (WTS), microarray, or specific RNA panel. The TMB score was obtained from original datasets if available; otherwise, the TMB score of a sample was defined by its total number of non-synonymous mutations. The information on objective response and progress-free survival (PFS) was obtained from original studies, of which 25 were evaluated by the Response Evaluation Criteria In Solid Tumors (RECIST) v1.1, one was by the immune-related RECIST (irRECIST), and one was self-defined. Overall survival (OS) was also obtained from original studies. The OS of three in-house datasets was defined as the time from the treatment date to the date of death or the end of follow-up.

**Data processing**

All clinical and molecular data were converted into a uniform format. For transcriptomic data, the fragmentation per kilobase million (FPKM) was converted to the transcript per million bases (TPM), and then all the TPM was standardized with log_2_(TMP + 0.001). Read counts were converted into log_2_(CPM + 0.001). Signatures of immune and oncogenic pathways were obtained from a previously published literature [13] or MSigDB Database (<https://www.gsea-msigdb.org/gsea/msigdb>). Then the single sample gene set enrichment analysis (ssGSEA) algorithm was applied to calculate the enrichment scores of these signatures.

**Statistics**

Kaplan-Meier (KM) survival curves and univariable/multivariable Cox regression models were used to analyze the associations between biomarkers and OS/PFS. A random-effects meta-analysis model was used to pool the results from multiple studies with the “meta” and “survminer” R packages. The ggforest function in the “survminer” package was modified for forest plotting. Continuous variables between two groups were compared using the Mann-Whitney U test and categorical variables using the Chi-square test or Fisher exact test. The Spearman correlation analysis was used to explore the relationship between biomarkers and gene expression. *P* value for multiple tests was adjusted by the Benjamini-Hochberg in the correlation analysis module.

**Uploading function**

IMPACT allows users to upload their datasets. Uploaded data should include clinical outcome information and their related genetic and/or transcriptomic data. For somatic mutation, each patient should be annotated with mutation (MUT) or wild type (WT). Transcriptomic data should be normalized and transformed with log2. An example of the data format is provided on the “User-defined” page of IMPACT.

**Parameter customization**

The key parameters can be customized during analyses by users to fulfill their specific needs, such as variant-type defined mutation (MUT) and wild type (WT), cut-off values for continuous variable categorization, graph colors, and legend positions. These customizations can greatly increase the utility and flexibility of IMPACT and allow users to generate ready-to-use figures.
